# Bulgarian consumers’ objective understanding of front-of-package nutrition labels: a comparative, randomized study

**DOI:** 10.1186/s13690-020-00416-z

**Published:** 2020-06-09

**Authors:** Valentina A. Andreeva, Manon Egnell, Teodora Handjieva-Darlenska, Zenobia Talati, Mathilde Touvier, Pilar Galan, Serge Hercberg, Simone Pettigrew, Chantal Julia

**Affiliations:** 1 Nutritional Epidemiology Research Group (EREN), Sorbonne Paris Nord University/INSERM U1153/INRAE U1125/CNAM), Epidemiology and Statistics Research Centre - University of Paris (CRESS), 74 rue Marcel Cachin, 93017 Bobigny, France; 2 Department of Pharmacology and Toxicology, Medical University, 1431 Sofia, Bulgaria; 3 Bulgarian Association for the Study of Obesity and Related Disorders, 1233 Sofia, Bulgaria; 4 School of Psychology, Curtin University, Bentley, WA 6102 Australia; 5 Department of Public Health, Avicenne Hospital, 93017 Bobigny, France; 6 The George Institute for Global Health, Newtown, NSW 2042 Australia

**Keywords:** Front-of-package food label, Nutrition labeling, Diet, Eastern Europe, Public health

## Abstract

**Background:**

Bulgaria continues to lag behind other EU Member States with respect to chronic disease morbidity and mortality prevention.

**Methods:**

In line with efforts targeting the improvement of dietary practices, this comparative study assessed objective understanding of five different front-of-package labels (FOPL) (Reference Intakes, Multiple Traffic Lights, Warning label, Nutri-Score, and Health Star Rating) in a sample of 1010 Bulgarian adults. Objective understanding was assessed by comparing the results of two nutritional quality ranking tasks (with and without FOPL) in an online randomized experiment featuring three food categories (pizza, cakes, breakfast cereals). Multivariable ordinal logistic regression models within and across food categories were fit.

**Results:**

Compared with the Reference Intakes group, participants randomized to Nutri-Score exhibited the largest improvement in product ranking ability across food categories (OR = 2.33; 95% CI: 1.55–3.51), followed by those randomized to Health Star Rating (OR = 1.99; 95% CI: 1.32–3.00). Nutri-Score also performed best within two (pizza and breakfast cereals) of the three food categories. The Multiple Traffic Lights and Warning label groups did not display any significant improvement in objective understanding either within or across food categories compared with the Reference Intakes group.

**Conclusion:**

Nutri-Score, which is a summary, interpretive, polychromatic FOPL, emerged as the most effective model in the Bulgarian context, with the potential to help consumers better understand the nutritional quality of food. The findings are of particular interest to public health policymakers in the region and across Europe, as the debate about an EU-wide FOPL model continues to gather momentum.

**Trial registration:**

Registration number ACTRN12618001221246. Trial registered at the Australian New Zealand Clinical Trials Registry.

## Background

Fourteen years into its European Union (EU) membership, Bulgaria continues to lag behind other EU Member States with respect to curbing the prevalence of risk behaviors (smoking, unhealthy diets, alcohol use) and chronic disease morbidity, and with respect to life expectancy, which is about 6 years below the EU average [1–3]. For example, in the EU in 2017, the proportion of cardiovascular disease mortality varied from 23% in France to 60% in Bulgaria among men, and from 25% in Denmark to 70% in Bulgaria among women [2]. An unhealthy diet has been recognized as the largest behavioral contributor to premature cardiovascular mortality risk [2], and evidence about the role of nutrition in the development and progression of cancer is growing [4]. Regarding diabetes mellitus and obesity among adults, rates in Bulgaria are slightly below the EU average, yet have risen by more than 20% over the past decade [1, 5]. In October 2015, the Bulgarian Ministry of Health introduced a proposal for a public health tax law intended to steer the population towards healthy dietary choices, restrict production of food of low nutritional quality, and reduce long-term healthcare expenditures [6]. The proposal, which featured taxation of foods and beverages containing quantities of sugar, salt, trans-fatty acids, caffeine, or taurine exceeding pre-defined limits, was rejected by the National Assembly following a vigorous debate [6]. This outcome is alarming and could be viewed as reflecting not only opposition by food industry advocates, but also with respect to the broader context reflecting the population’s generally low level of awareness about chronic disease prevention. For example, evidence from a recent nationally representative survey showed that, among Bulgarian adults aged 20 years and older, knowledge about risk factors and causes of serious diseases was markedly insufficient and often inaccurate [7].

Globally, the deleterious consequences of fat/sugar-dense diets coupled with sedentariness have prompted the World Health Organization (WHO) to advance not only individual, but also industry-level recommendations, such as reformulating processed food products for the purpose of reducing fat/sugar/salt content and ensuring availability and affordability of nutritious food worldwide [8]. In that context, different algorithms for ranking food according to its nutritional composition have been developed by nutrient profiling experts. Front-of-package labels (FOPL) are a means of conveying such ranking information to consumers. Since 1989, when the first FOPL - the “Green Keyhole symbol” - was launched in Sweden, various FOPL models have been developed and numerous countries (not Bulgaria at present) have introduced voluntary or mandatory FOPL systems for pre-packaged food and beverage products [9].

Broadly, FOPL fall into nutrient-specific or summary indicators, with the former featuring numeric data about nutrient content/quantity, while the latter combine several elements into graphical and/or color-coded indicators [10]. Recent experimental work showed that use of FOPL could lead to healthier food purchases, with reduced quantities of sugar, sodium, saturated fat, and/or calories [11, 12], and a literature review of randomized studies has reported an estimated 18% increase in the proportion of consumers choosing a healthy food product in the presence of FOPL [13]. Indeed, a 2018 WHO report highlighted the fact that FOPL that are noticeable and easily understandable have the potential to urge consumers to make informed healthier food choices and drive product reformulation by manufacturers [14].

Given the urgent need for public health research and intervention efforts aimed at improving food-related knowledge, health behaviors and outcomes in Bulgaria, the present comparative study, which is part of a 12-country experimental research project on the effectiveness of various FOPL [15], assessed objective understanding of five different FOPL in a quota-based sample from the Bulgarian population.

## Methods

### Participants

This analysis utilized data from Bulgarian adults (*N* = 1013, aged 18 years and over), obtained between April and July 2018 for a multinational (Argentina, Australia, Bulgaria, Canada, Denmark, France, Germany, Mexico, Singapore, Spain, the UK, and the US) Internet-based randomized experiment on FOPL, described in detail elsewhere [15]. Briefly, the same ISO-accredited international web panel provider called PureProfile was used for the recruitment of participants across the 12 countries. PureProfile (https://business.pureprofile.com/) is headquartered in New South Wales, Australia, and is one of the web’s oldest survey panel providers, working with brands, publishers and research groups worldwide. It employs direct-to-consumer technology platforms and recruits survey participants via online and mass media advertising and word-of-mouth referrals. For the present study, PureProfile recruited ~ 1000 participants per country using quota sampling based on age (one-third in each of the following age categories: 18–30, 31–50, and > 51 years), sex (50% women), and socio-economic status (SES) (one-third in each of the following household income categories: low, medium [falling within a 33% bracket around the country-specific median], and high).

All volunteers provided informed consent prior to enrollment. The study protocol was approved by the Institutional Review Board of the French Institute for Health and Medical Research and by Curtin University Human Research Ethics Committee. The trial is registered with the Australian New Zealand Clinical Trials Registry (http://www.ANZCTR.org.au/ACTRN12618001221246.aspx). This study is reported according to the CONSORT guidelines.

### Trial objectives

The principal aim of the trial was to compare the effectiveness of five different FOPL (described below) in terms of helping individuals understand the relative nutritional quality of different types of food. The present analysis is focused on objective (also referred to as substantial) understanding of FOPL, defined as the consumer’s ability to correctly interpret the information provided by a FOPL (ie, as intended by its designers) [16].

### FOPL tested in trial

Five FOPL, which appear on packaged processed food/beverages in retail environments in various countries, were tested in this trial: three nutrient-specific schemes (Reference Intakes [RI], Multiple Traffic Lights [MTL], and Warning label) and two summary indicators (Health Star Rating [HSR] and Nutri-Score).

RI, promoted by Europe’s food and drink industry, replaced Guideline Daily Amounts in 2014. This nutrient-specific, monochrome scheme provides numeric information per portion and per 100 g/ml regarding the quantity and proportion (with respect to the recommended intake for an average adult) of calories, total and saturated fat, sugar, and salt [17]. MTL is also a nutrient-specific FOPL; it has been available since 2005 and was endorsed by the UK government in 2013. It combines RI and color-coding of the quantity of energy and specific nutrients (total and saturated fat, sugar, salt) contained in a single portion. Red, amber, and green colors represent low, medium, and high amounts of each of these nutrients (without representing dietary claims), and are calculated according to nutrient-specific thresholds per 100 g/ml. For example, in order to feature green for total fat, a given food product must not contain > 3 g of fat per 100 g [18]. Like RI (when used in Europe), MTL is provided on a voluntary basis and comprises numeric information about energy, total and saturated fat, total carbohydrates, sugars, protein, and salt [19]. The third nutrient-specific FOPL - Warning label - was first introduced in 2016 in Chile on a mandatory basis and is now part of the Chilean Food Labeling and Marketing Law [20]. It is a monochrome indicator that flags food products containing large amounts of energy or specific nutrients (eg, saturated fat, sugar, salt) implicated in chronic disease risk [20]. Since its initial introduction, similar Warning labels have been approved for implementation in other countries, such as Uruguay, Peru, and Israel [20].

HSR is a monochrome, scaled, summary indicator which was introduced in Australia and New Zealand in 2014. It features nutrient-specific information and ratings from 0.5 to 5 stars intended to help consumers identify healthier food/beverage options within a given product range. HSR, which uses an algorithm adapted from the British Food Standards Agency Nutrient Profiling System [21], is a government-endorsed nutrient profiling model that was designed to complement general dietary guidance [22]. The second summary indicator - the five-color, graded Nutri-Score - was first implemented in France in 2017 [23]. Its algorithm is intended to reflect overall nutritional quality and is also largely based on the one developed by the British Food Standards Agency Nutrient Profiling System [21], following minor modifications by the High Council for Public Health in conformance with French dietary guidelines regarding cheese products, added fats, and beverages [24]. Nutri-Score is calculated per 100 g, taking into account energy, saturated fat, sugar, sodium, fiber, protein, and proportion of fruit/vegetables/nuts [25]. Using nutrient-specific thresholds, five levels of nutritional quality are derived, ranging from dark green (letter A, highest nutritional quality) to red (letter E, lowest nutritional quality). The FOPL features the entire scale, with the color/letter corresponding to the product’s nutritional quality enlarged [26]. Since its initial implementation in France, it has been adopted in other countries, such as Spain, Belgium, and Germany.

**Fig. 1:**
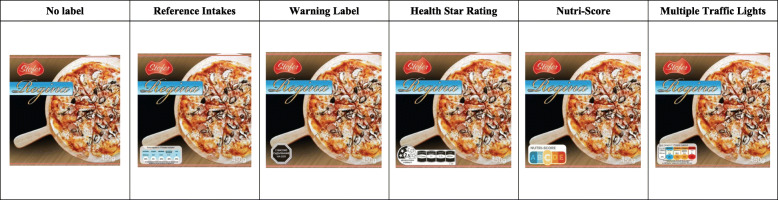
Examples of images without a label and with each of the five front-of-package labels tested in the study

Figure 1 presents an example of all five FOPL as displayed on one of the products (pizza) featured in the trial. Each FOPL appeared in the same position on any given product, covering roughly the same surface area on the package. None of the tested FOPL is currently implemented in Bulgaria.

### Trial design

For this trial, mock food packages were created to resemble real food products belonging to three distinct categories: breakfast cereals, pizzas, and cakes. All products belonged to the fictional brand *Stofer*, thus preventing familiarity or purchasing habits from interfering with product evaluation. Within each food category, a set of three products with distinct nutritional profiles (lower, intermediate, and higher nutritional quality) were presented; the same food products were shown across the various FOPL conditions (described below). No other nutrition-related information or food quality indicators (e.g., organic certification) were provided.

Participants were asked to perform two tasks in an experiment conducted online. For the first task, they were sequentially presented with images of three sets of three FOPL-free products (one set of three kinds of pizza, one set of three types of cake, and one set of three types of breakfast cereal) and were asked to rank each product according to its nutritional quality. Ranking options included: “1 = Highest nutritional quality,” “2 = Medium nutritional quality,” and “3 = Lowest nutritional quality.”

Upon completion of the first task, participants were randomized by the web panel provider to one of five FOPL conditions - HSR, MTL, Nutri-Score, Warning label, and RI (modeled as the reference condition) - and then asked to repeat the ranking task, which featured images of the same three sets of three products, each displaying one of the five FOPL. Participants were unaware that they would be seeing the products twice, or that FOPL would be included the second time. Potential presentation order effects were minimized by randomizing the order in which the food categories and the images of the respective products appeared on the screen. For process evaluation purposes, at the end of the trial, participants were asked whether they recalled having seen the FOPL to which they were exposed.

### Dependent variable

The main outcome was objective understanding of FOPL, which was assessed by comparing the results of the two ranking tasks. Specifically, we assessed participants’ ability to use the nutrition information conveyed by FOPL to correctly rank food products according to their nutritional quality. We expected that results of the second ranking task would be superior to those of the initial, FOPL-free ranking task, due to the provision of nutrition information.

### Covariates

Upon enrollment in the trial, participants completed a short online questionnaire, providing information about their age, sex, household income, educational level (up to high school, trade certification or equivalent, undergraduate degree, graduate degree), presence of children < 14 years in the household, grocery shopping responsibility (yes, no, shared), knowledge about nutrition (none, very limited, average, extensive) and perceived quality of their own diet (very unhealthy, mostly unhealthy, mostly healthy, very healthy). Self-reported annual household income in Bulgarian leva was assigned to one of three categories (low, medium, high) using 2017 data on the Bulgarian median income (3590 €, exchange rate = 1.735) from the World Income Inequality Database [27].

### Statistical analyses

Individuals who reported never purchasing ≥2 of the three food product categories were ineligible for the study. Likewise, if a person reported never purchasing products from a given food category, his/her responses to the corresponding ranking task were excluded. Descriptive characteristics of the sample are reported as percentages obtained from chi-squared tests or as mean (SD) obtained from Student *t* tests. For each participant, we computed the change (expressed in percent) in the number of correct responses within and across the three food categories. Ranking was considered correct if all three products were ranked in the expected order (ie, no partially correct ranking). For each food category, raw scores ranged between − 1 (deterioration) and + 1 (improvement), with a score of 0 signifying no change. These scores were then summed up across the three food categories, thus arriving at a final global score per task ranging from − 3 to + 3. Multivariable ordinal logistic regression models (RI = reference) were fit within and across food categories in order to assess the association of FOPL exposure with change in product ranking ability. These models were adjusted for sex, age, education, household income, children < 14 years living in household, grocery shopping responsibility, self-assessed diet quality, and knowledge about nutrition. Odds ratios (OR), 95% confidence intervals (CI), and *p*-values corrected via the false discovery rate method to account for multiple comparisons are reported. Potential effect modification by sex, age group, education and income level was assessed via individual tests for interaction. Finally, to test the robustness of the main findings, a sensitivity analysis excluding participants who did not recall having seen a FOPL was also carried out.

All statistical analyses were performed using SAS version 9.4 (SAS Institute Inc., Cary, NC, USA). A significance level of 0.05 was used.

## Results

From the initial sample of 1013 adult volunteers, we excluded 3 individuals due to aberrant SES data. Thus, the final sample included 1010 participants, 10 of whom reported never purchasing pre-packaged cakes, 53 - never purchasing pre-packaged pizza, and 49 - never purchasing breakfast cereals. The mean age was 39.2 ± 13.2 years (range 18–79 years) and women represented 49.9% of the sample. Effect modification (ie, interaction) was not statistically significant (sex *p* > 0.37; age group *p* > 0.23; educational level *p* > 0.24; income level *p* > 0.65).

Participant characteristics by FOPL condition are presented in Table 1.

**Table 1:** Descriptive characteristics of participants by randomization group (*N* = 1010)

	Multiple Traffic Lights		Health Star Rating		Nutri-Score		Warning Label		Reference Intakes	
Sex										
Male	101	(49.7)	105	(52.0)	110	(54.5)	91	(45.1)	99	(49.3)
Female	102	(50.3)	97	(48.0)	92	(45.5)	111	(54.9)	102	(50.7)
Age, *y*										
18–30	64	(31.5)	70	(34.7)	71	(35.2)	69	(34.2)	83	(41.3)
31–50	87	(42.9)	74	(36.6)	79	(39.1)	74	(36.6)	65	(32.3)
51+	52	(25.6)	58	(28.7)	52	(25.7)	59	(29.2)	53	(26.4)
Mean (SD)	38.9	(12.8)	39.9	(13.1)	39.1	(13.2)	39.7	(13.6)	38.4	(13.3)
Education										
Up to high school	34	(16.8)	29	(14.4)	25	(12.4)	30	(14.9)	27	(13.4)
Trade certification or equivalent	52	(25.6)	52	(25.7)	61	(30.2)	38	(18.8)	49	(24.4)
Undergraduate degree	49	(24.1)	54	(26.7)	53	(26.2)	59	(29.2)	47	(23.4)
Graduate degree	68	(33.5)	67	(33.2)	63	(31.2)	75	(37.1)	78	(38.8)
Household income										
Low	50	(24.6)	62	(30.7)	62	(30.7)	51	(25.3)	56	(27.9)
Medium	77	(37.9)	58	(28.7)	71	(35.1)	74	(36.6)	79	(39.3)
High	76	(37.5)	82	(40.6)	69	(34.2)	77	(38.1)	66	(32.8)
Children ≤14 y in household										
No	129	(63.6)	137	(67.8)	132	(65.3)	137	(67.8)	124	(61.7)
Yes	74	(36.4)	65	(32.2)	70	(34.7)	65	(32.2)	77	(38.3)
Grocery shopping responsibility										
No	11	(5.4)	18	(8.9)	9	(4.5)	13	(6.4)	13	(6.5)
Shared	71	(35.0)	74	(36.6)	79	(39.1)	57	(28.2)	68	(33.8)
Yes	121	(59.6)	110	(54.5)	114	(56.4)	132	(65.4)	120	(59.7)
Knowledge about nutrition										
None or very limited	31	(15.3)	52	(25.7)	51	(25.2)	38	(18.8)	44	(21.9)
Average level	138	(68.0)	119	(58.9)	112	(55.5)	132	(65.4)	126	(62.7)
High level	34	(16.7)	31	(15.4)	39	(19.3)	32	(15.8)	31	(15.4)
Self-assessed diet quality										
Very unhealthy	7	(3.5)	9	(4.5)	13	(6.4)	11	(5.5)	8	(4.0)
Mostly unhealthy	117	(57.6)	123	(60.9)	124	(61.4)	119	(58.9)	124	(61.7)
Mostly or very healthy	79	(38.9)	70	(34.6)	65	(32.2)	72	(35.6)	69	(34.3)

Values refer to number (%) except when noted otherwise

Having children < 14 years in the household, having a graduate degree, high household income, a mostly/very healthy diet, and shared grocery shopping responsibility were respectively reported by about a third of participants. Across FOPL conditions, approximately two-thirds of participants reported an average level of knowledge about nutrition.

### Effect of FOPL condition on product rankings

The multivariable ordinal logistic regression results are summarized in Table 2.

**Table 2:** Objective understanding of FOPL as measured by change in correct food product ranking before (without FOPL) and after randomization (with FOPL)

Food category	N	Health Star Rating		Multiple Traffic Lights		Nutri-Score		Warning Label	
		OR (95% CI)	*p*	OR (95% CI)	*p*	OR (95% CI)	*p*	OR (95% CI)	*p*
All categories	1010	1.99 (1.32–3.00)	*0.003*	1.14 (0.76–1.70)	*0.62*	2.33 (1.55–3.51)	*< 0.001*	1.29 (0.86–1.93)	*0.32*
	699^a^	2.31 (1.39–3.83)	*0.002*	1.16 (0.73–1.86)	*0.53*	2.46 (1.56–3.88)	*< 0.001*	1.24 (0.77–2.02)	*0.51*
Pizzas	957	1.77 (1.09–2.88)	*0.05*	0.98 (0.61–1.58)	*0.93*	2.37 (1.45–3.87)	*0.003*	1.36 (0.84–2.21)	*0.32*
Cakes	1000	3.04 (1.74–5.33)	*< 0.001*	1.57 (0.90–2.72)	*0.20*	2.90 (1.66–5.08)	*0.003*	1.67 (0.96–2.91)	*0.14*
Breakfast cereals	961	1.46 (0.78–2.75)	*0.32*	1.33 (0.71–2.50)	*0.46*	1.97 (1.06–3.66)	*0.07*	0.92 (0.48–1.73)	*0.84*

Multivariable ordinal logistic regression (“Reference Intakes” = reference) with adjustment for sex, age, education, household income, children < 14 y living in household, grocery shopping responsibility, self-assessed diet quality, and knowledge about nutrition; all *p*-values corrected via false discovery rate to account for multiple comparisons

*CI* confidence interval, *FOPL* front-of-package label, *OR* odds ratio

^a^Sensitivity analysis excluding 311 individuals who did not recall seeing FOPL on products

In the analysis combining all three food categories, compared with the RI group, participants randomized to Nutri-Score exhibited the largest improvement in product ranking ability (OR = 2.33; 95% CI: 1.55–3.51), followed by those randomized to HSR (OR = 1.99; 95% CI: 1.32–3.00). In turn, compared with RI, the MTL and Warning label groups did not display any significant improvement in objective understanding across food categories. Favorable change in product ranking ability (ie, objective understanding of FOPL) was significantly and positively associated with grocery shopping responsibility (exclusive or shared), but was unrelated to sex, age, SES, children < 14 years living in household, diet quality, or knowledge about nutrition.

For the pizza category, the largest improvement in product ranking ability relative to RI was again observed in the Nutri-Score group (OR = 2.37; 95% CI: 1.45–3.87), followed by the HSR group (OR = 1.77; 95% CI: 1.09–2.88). The results were reversed for the cakes category, where HSR slightly outperformed Nutri-Score (OR = 3.04; 95% CI: 1.74–5.33 versus OR = 2.90; 95% CI: 1.66–5.08). Finally, for the breakfast cereals category, none of the FOPL conditions resulted in statistically significant associations with product ranking ability. In that analysis, only the Nutri-Score group exhibited marginal statistical significance (OR = 1.97; 95% CI: 1.06–3.66; *p* < 0.07), following correction of the significance level to account for multiple comparisons. The MTL and Warning symbol groups did not display any statistically significant associations with product ranking ability for any of the food categories.

In order to test the robustness of the main findings across food categories, we performed a sensitivity analysis following the exclusion of 311 participants who did not recall having seen a FOPL during the trial. Results of this sensitivity analysis were consistent with the main results. Specifically, compared with the RI group, participants randomized to Nutri-Score exhibited an even larger improvement in product ranking ability (OR = 2.46; 95% CI: 1.56–3.88), followed by those randomized to HSR (OR = 2.31; 95% CI: 1.39–3.83). Associations with product ranking ability in the MTL and Warning label groups remained statistically non-significant.

## Discussion

This experimental, comparative study demonstrated marked differences in the objective understanding of FOPL according to graphic format and - to a lesser extent - food category, irrespective of the individuals’ SES profiles. Supporting the main hypothesis, the trial results provided evidence that the nutrition information conveyed by FOPL could augment consumers’ ability to correctly rank food products according to their nutritional quality. Specifically, among the five FOPL conditions - HSR, MTL, Nutri-Score, Warning label, and RI (reference) - participants randomized to Nutri-Score exhibited the largest improvement in product ranking ability across food categories. These associations were seen both in the main and sensitivity analyses. Nutri-Score also performed best within two (pizza and breakfast cereals) of the three food categories, followed by HSR. In the cakes category, however, the HSR group significantly outperformed the Nutri-Score group. In turn, compared with RI, the MTL and Warning label groups did not display any significant improvement in objective understanding either within or across food categories.

The present study, which is part of a 12-country experimental research project on the effectiveness of various FOPL [15], demonstrated some universal as well as some country-specific effects. For example, in all 12 countries and across all three food categories, Nutri-Score performed best [15]. Interestingly, Nutri-Score showed greater effectiveness compared with other FOPL, even in countries, such as the UK and Australia, where alternative official FOPL have been implemented (eg, MTL, HSR) [15]. Next, in 11 out of the 12 countries, the Warning label did not appear to play a role in product ranking ability, displaying a significant association only among consumers in Singapore [15]. In contrast, MTL elicited significant favorable changes in consumers’ product ranking ability in 8 out of the 12 countries (non-significant associations seen in the samples from Bulgaria, Argentina, Australia, and Denmark) [15]. Regarding HSR, significant effects were observed only in Bulgaria, Australia, and Singapore [15]. Prior international FOPL research has suggested that interest in healthy eating on the population level, the length and intensity of the public discourse about nutrition and food labeling, and media-generated familiarity might help explain country-specific effects of FOPL [16, 28].

The present findings among Bulgarian consumers suggest that summary indicators (Nutri-Score, HSR) have a clear advantage over nutrient-specific schemes (MTL, Warning label, RI) with respect to consumers’ objective understanding of FOPL. Prior research has revealed that consumers favor simple FOPL that entail a relatively low cognitive workload and rapid processing, especially considering that point-of-purchase decisions are made quickly [29]. Whereas both Nutri-Score and HSR are summary indicators, the superior performance of the former might be attributed to its polychromatic design, especially as it features a green-to-red scale [30]. Indeed, eye-tracking research has associated color-coding with faster detection and attention to nutrition labels, in particular among consumers who did not have explicit nutritional goals [31]. Further, research with German and Polish consumers provided evidence that color-coding increased perceived capability of making healthful choices [28].

To the best of our knowledge, a comparison among FOPL (MTL, Nutri-Score, and RI) was first discussed in Bulgaria in 2017 in a local publication sponsored by the Food Industry Union and the Bulgarian Food Safety Agency [32]. Next, at the start of the Bulgarian presidency of the EU in 2018, Nutri-Score was re-introduced to the public along with the extensive scientific validation and decision-making process leading to its eventual adoption in several EU countries [33]. In Bulgaria, unhealthy diets (despite having official dietary guidelines for adults since 2006), vast regional SES inequalities, insufficient public health measures, and ineffective legislative efforts to mitigate chronic disease risk persist [1, 3, 34]. For example, results are still pending regarding the effectiveness of two salt-reduction efforts: a nationwide information campaign for salt reduction launched in 2010 by the Bulgarian Ministry of Health and a WHO-supported salt reduction initiative launched in 2013 and targeting five of the principal food groups in the country [35]. In addition to the mandatory EU nutrition declaration requirement introduced in December 2016 (regarding energy, total and saturated fat, total carbohydrates, sugars, protein, and salt per 100 g/ml) [36], it appears necessary to add FOPL to the national policy agenda. It has been suggested that unlike other label formats, color-coded FOPL, such as Nutri-Score, could convey nutrition-related information across population strata [31]. Unlike evidence in other populations, this study revealed that favorable change in product ranking ability (ie, objective understanding of FOPL) among Bulgarian consumers was unrelated to sex, age, SES, diet quality, or knowledge about nutrition [16]. Finally, Nutri-Score has been shown to have a significant, favorable impact on portion size selection [37] and on the nutritional composition of food purchases from an experimental Web-based supermarket [38].

Two limitations of this work pertain to the use of an online panel with quota-based rather than representative sampling, and the study being conducted as an online experiment rather than an actual grocery shopping experience where numerous factors (familiarity, brand loyalty, access to information about ingredients/nutritional composition, time pressure) likely play a role in perceptions and choice. It has been estimated that attention to nutrition labels lasts between 25 and 100 milliseconds [39]. In turn, a notable strength of the trial was the use of three sets of three products, which approximated real-life situations while decreasing the possibility of correct responses by chance. The food categories (pizza, cakes, and breakfast cereals) were chosen as stimuli because they are consumed in all 12 countries included in the trial and because there is marked variability in the nutritional quality within each category [15]. During the trial, any potential learning effects were mitigated by means of randomizing the presentation order within sets and across food categories.

## Conclusions

The WHO European Food and Nutrition Action Plan 2015–2020 has highlighted the introduction of interpretive, consumer-friendly FOPL as a priority policy issue [14]. In the present comparative study, Nutri-Score, which is a summary, interpretive, polychromatic FOPL officially used in France, Spain, Belgium, and Germany, emerged as the most effective model in the Bulgarian context where no government-mandated FOPL exists at present. In addition, among the reviewed 121 policy interventions targeting healthy eating in Europe, only 4 pertained to nutrition labeling and none took place in Eastern Europe [40]. Therefore, the findings of the present study are of particular interest to public health policymakers in the region and across Europe, as the debate about an EU-wide FOPL model continues to gather momentum.

## Data Availability

The data supporting the results of this study are included in the present article or in the supplementary material.

## Abbreviations

CI: Confidence interval
EU: European Union
FOPL: Front-of-package label
HSR: Health Star Rating
MTL: Multiple Traffic Lights
OR: Odds ratio
RI: Reference Intakes
SES: Socio-economic status
WHO: World Health Organization
